# Enhanced Dye Adsorption on Cold Plasma-Oxidized Multi-Walled Carbon Nanotubes: A Comparative Study

**DOI:** 10.3390/nano14151298

**Published:** 2024-08-01

**Authors:** Anastasia Skourti, Stefania Giannoulia, Maria K. Daletou, Christos A. Aggelopoulos

**Affiliations:** 1 Laboratory of Cold Plasma and Advanced Techniques for Improving Environmental Systems, Institute of Chemical Engineering Sciences, Foundation for Research and Technology Hellas (FORTH/ICE-HT), 26504 Patras, Greece; 2 Laboratory of Advanced Materials and Electrochemical Energy Conversion Devices, Institute of Chemical Engineering Sciences, Foundation for Research and Technology Hellas (FORTH/ICE-HT), 26504 Patras, Greece

**Keywords:** carbon nanotubes, cold plasma, surface oxidation, water treatment, adsorption process, dyes

## Abstract

The oxidation of multi-walled carbon nanotubes (MWCNTs) using cold plasma was investigated for their subsequent use as adsorbents for the removal of dyes from aqueous solutions. The properties of MWCNTs after plasma modification and their adsorption capacities were compared with pristine and chemically oxidized nanotubes. The modification process employed a reactor where plasma was generated through dielectric barrier discharges (DBD) powered by high-voltage nanosecond pulses. Various modification conditions were examined, such as processing time and pulse voltage amplitude. The degree of oxidation and the impact on the chemistry and structure of the nanotubes was investigated through various physicochemical and morphological characterization techniques (XPS, BET, TEM, etc.). Maximum oxidation (O/C = 0.09 from O/C = 0.02 for pristine MWCNTs) was achieved after 60 min of nanopulsed-DBD plasma treatment. Subsequently, the modified nanotubes were used as adsorbents for the removal of the dye methylene blue (MB) from water. The adsorption experiments examined the effects of contact time between the adsorbent and MB, as well as the initial dye concentration in water. The plasma-modified nanotubes exhibited high MB removal efficiency, with adsorption capacity proportional to the degree of oxidation. Notably, their adsorption capacity significantly increased compared to both pristine and chemically oxidized MWCNTs (~54% and ~9%, respectively). Finally, the kinetics and mechanism of the adsorption process were studied, with experimental data fitting well to the pseudo-second-order kinetic model and the Langmuir isotherm model. This study underscores the potential of plasma technology as a low-cost and environmentally friendly approach for material modification and water purification.

## 1. Introduction

Water pollution has increased over the past decades due to factors such as industrial growth, intensified agriculture, and inadequate waste management practices. These human activities release pollutants into water bodies, impacting ecosystems and human health. One of the most common categories of water pollution is dyes, with textile industries playing the main contributing role [1]. The presence of dyes in water streams has significant effects on the environment and living organisms. Dyes are challenging to remove from water due to their complex chemical structures and resistance to conventional treatment methods. Their excellent solubility, non-biodegradability, and high stability of dyes in water make their removal a significant concern. Various techniques are being explored for treating dye-containing wastewater, with a focus on adsorption. Adsorption involves using materials like activated carbon or modified clay to attract and remove dye molecules and remove them from water [2,3,4,5,6,7].

Carbon nanotubes (CNTs) exhibit exceptional adsorption properties and are being intensively researched for the adsorption and removal of dyes from water due to their important advantages such as high stability, high surface area, tunable surface properties, and low cost [7,8,9]. The widespread and rapidly increasing use of CNTs in scientific and technical applications and their incorporation into industrial products have raised numerous research questions about their potential toxicity to humans and the environment [10]. Despite the challenges, their multifunctional properties continue to drive research and innovation. The reusability of CNT-based adsorbents is important for the cost as well as the environmental perspective [11]. Consequently, research strives to understand the adsorption conditions of CNTs in different scenarios.

It is well known that the hydrophobic nature of CNTs limits their applications. Their surface modification is often required for enhancing their compatibility with other materials, controlling their interactions and/or reactivity, and improving their dispersion in media [8,12,13]. Functional groups on carbon surfaces are commonly bonded with heteroatoms. These functional groups are traditionally categorized based on the heteroatoms attached to the carbon surface, including oxygen- [14], fluoro- [15], nitrogen- [16,17,18], sulfur- [17,19], and hydrogen-containing [20] functional groups. The most studied surface modification of CNTs is the conventional oxidation method using strong chemical acids such as HCl, HNO_3_, piranha H_2_SO_4_/H_2_O_2_ mixture, and NH_4_OH/H_2_O_2_ mixture [21,22,23]. It is known that treating CNTs with chemicals causes significant alterations in their structure, especially when the original CNTs have a high number of defects [8,21]. These oxidation methods also have significant disadvantages, such as the reduction in the specific surface area and total pore volume, as well as the production of toxic chemicals and their gases that lead to environmental pollution [24]. Moreover, as a treatment, it is considered an expensive process, complicated, and time-consuming, with significant energy consumption [25].

An alternative method for modifying materials is cold plasma technology, a ‘green’ approach, introducing functional groups on the surface of CNTs [26,27]. Plasma can be generated at either low pressure or atmospheric pressure by supplying energy to a gaseous medium through mechanical, thermal, or chemical methods, as well as via radiation, nuclear energy, or the application of a voltage or electromagnetic waves [28]. Cold atmospheric pressure plasma serves as a unique source of various active species and particles, including free radicals, ions, excited atoms and molecules, metastable atoms, and UV radiation [29]. Cold plasma’s distinctive features, including eco-friendliness, fast processing, mild reaction conditions, and low process cost, have made it widely applicable in many scientific and technological domains. Despite materials’ modification, cold plasma finds applications in the food industry with an emphasis on disinfection [30], as well as air, water, and soil decontamination through the rapid and efficient degradation of organic pollutants [31,32]. In the biomedical field, it can contribute to the inactivation of bacteria through disinfection and sterilization [33].

Concerning surface functionalization of materials, plasma treatment times are shorter than multi-hour chemical treatment, and a wide range of functional groups are available depending on the plasma parameters such as pressure, power, gas type, gas flow rate, treatment time, sample layout, and reactor type [34]. It has been reported that surface modification involving the introduction of nitrogen functional groups into polystyrene has been carried out in reactors powered by both pulsed high voltage and microwaves [35]. Additionally, other studies have demonstrated that cold plasma can introduce functional groups such as -COOH, -OH, -COOR, >C=O, and -NH_2_ onto the surfaces of certain polymers for diverse applications [36]. Daletou and Aggelopoulos investigated the surface oxidation of multi-walled carbon nanotubes (MWCNTs) by the use of nanosecond pulsed dielectric barrier discharge (DBD) cold plasma and compared the induced properties with chemically treated (with HNO_3_) samples [26]. Plasma treatment successfully oxidized the surface by introducing oxygen-bearing (C-O and C=O) moieties with no notable changes in the graphitic structure, thus validating a dry, one-step procedure with a very short process time (~10 min) and an energy efficiency of ~200 g MWCNT/kWh. Plasma predominantly introduced C-O functional groups. However, over time and depending on the conditions, the proportion of C=O groups increased to levels comparable to those achieved through chemical oxidation. A comparison of MWCNTs after air–DBD plasma oxidation and chemical treatment was also carried out by Naseh et al. [24]. It was proven that plasma oxidation caused less variation in the nanotube structure in contrast to the chemical treatment. Also in this study, it was observed that carboxyl groups formed more readily in the chemically treated nanotubes, while plasma-treated nanotubes predominantly exhibited the formation of carbonyls and ethers [24]. In another attempt, Yu et al. used oxygen radiofrequency plasma to oxidize MWCNTs and thus improve their adsorption capacity of lead (II) from water [37]. The plasma-modified CNTs exhibited enhanced removal of lead (II). This improvement was attributed to several factors provoked by oxidation, including the increase in specific surface area, the formation of surface defects, the introduction of oxygen-containing functional groups, and better dispersion in water. In this study, the modification of MWCNTs using nanopulsed-DBD plasma was investigated for their potential application as adsorbents for removing Methylene Blue (MB) from aqueous solutions. MB is a thiazine cationic dye, commonly found in wastewater, stable in air and its aqueous solution. The properties and adsorption capacities of the plasma-modified MWCNTs were compared with those of unmodified (pristine) and chemically oxidized nanotubes treated with nitric acid. Various modification conditions, including treatment time and pulse voltage amplitude, were explored. The degree of oxidation and its effects on the nanotubes’ chemistry and structure were examined using a range of physicochemical and morphological characterization techniques, such as X-ray photoelectron spectroscopy (XPS), nitrogen adsorption porosimetry (BET), transmission electron microscopy (TEM), thermogravimetric analysis (TGA), and zeta potential measurements. The findings demonstrated that plasma-modified nanotubes could effectively remove MB from water, highlighting the advantages of plasma technology in these processes and applications.

## 2. Experimental Section

### 2.1. Materials

Pristine multi-walled carbon nanotubes (MWCNTs) with a purity of 97% and a diameter of 10–30 nm were purchased from Nanothinx S.A. (Rio, Greece). The dye used, Methylene Blue (MB) (C_16_H_18_CIN_3_S•2H_2_O, M.W.: 373.90 g/mol), was purchased from Sigma-Aldrich (St. Louis, MO, USA). Compressed dry air, supplied by Linde (Athens, Greece), was used as the plasma feeding gas. Triple distilled water (3D) was used for the preparation of all solutions.

### 2.2. Chemical Modification of MWCNTs

The chemical modification of the nanotubes was performed as follows: 1 g of MWCNTs was dispersed in 100 mL of nitric acid (HNO_3_ 65 wt%) in a flask with a condenser. The suspension was magnetically stirred under reflux conditions for 48 h. The resulting solid was extensively washed with water until the solution reached a neutral pH, filtered, and finally dried under vacuum at 80 °C.

### 2.3. Cold Plasma Experimental Setup and Experimental Conditions for the Modification of MWCNTs

**Figure 1:**
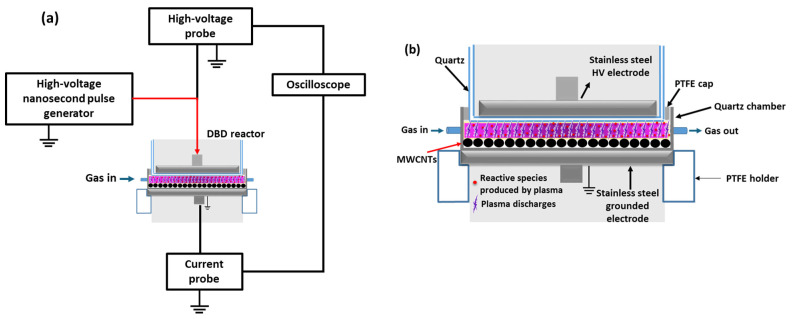
(**a**) Schematic representation of the experimental plasma setup used for the oxidation of MWCNTs and (**b**) details of the plate-to-plate DBD reactor.

The experimental setup for treating MWCNTs with nanopulsed-DBD, along with a detailed representation of the plasma reactor, is illustrated in Figure 1. High-voltage (HV) nanosecond pulses were used to energize a plate-to-plate dielectric barrier discharge (DBD) plasma reactor operating under atmospheric pressure using a suitable power supply (NPG-18/3500). This power supply can generate positive high-voltage pulses with a very short pulse rise time (~4 ns). During treatment, the applied high-voltage pulses and the circuit current were measured simultaneously using a high-voltage probe (Tektronix P6015A, 75 MHz, Beaverton, OR, USA) and a current monitor probe (Pearson electronics 2877, 300 Hz–200 MHz, Palo Alto, CA, USA), respectively. Their waveforms were monitored with a digital oscilloscope (Rigol MSO2302A, 300 MHz, 2 GSamples/s, Beijing, China). At a pulse voltage of 31.0 kV, the measured peak pulse current reached approximately 65 A, resulting in a significantly high peak instantaneous power of ~2 MW. However, due to the low duty cycle, the average power dissipated in the DBD reactor remained very low, at just 0.95 W [26,38]. Details regarding the calculation of the power dissipated in the DBD reactor and process energy efficiency can be found in previous studies [38,39].

The high-voltage electrode was a stainless-steel disk (40 mm diameter) covered with a quartz dielectric barrier (46 mm diameter and 2 mm bottom thickness), serving as the first dielectric (dielectric 1) of the discharge. The grounded electrode was a stainless-steel disk (68 mm diameter) fixed to the top of a PTFE cylinder/receptacle. Pristine MWCNTs were evenly spread (~1 mm thickness) at the bottom of a quartz reaction chamber (70 mm diameter and 2 mm bottom thickness), which served as the second dielectric (dielectric 2) of the discharge. The inter-electrode gap was set to ~7 mm, with the distance between the first dielectric surface and MWCNT’s surface being ~2 mm.

During treatment, the space above the MWCNTs was filled with plasma treatment gas (dry air), which flowed steadily over the sample surface, ensuring uniform plasma exposure. The air flow was kept constant at 1 L/min, controlled by a gas flow controller (Aalborg GFC17, Aalborg, Denmark). Plasma treatments were conducted with two different amplitudes of high-voltage pulses (23 and 31 kV), while the pulse repetition rate was set to 200 Hz. In each experiment, 50 mg of MWCNTs were treated in the DBD reactor for various durations (10, 20, 30, and 60 min).

### 2.4. Physicochemical Characterization of MWCNTs

After surface modification of the MWCNTs by cold plasma or chemical oxidation, the samples were characterized using various techniques to study the degree of oxidation, the types of oxygen groups introduced, and their morphology and structure compared to commercial pristine MWCNTs. Characterization by X-ray photoelectron spectroscopy (XPS) was particularly important for this study, as it provides insights into the surface chemistry and oxidation degree of the nanotubes. XPS experiments were conducted in a high vacuum system (P < 10^−9^ mbar) at room temperature using a SPECS (LHS-10) hemispherical electron analyzer and non-monochromatized MgKα radiation [40]. The XPS core level spectra were collected with the commercial software SpecsLab Prodigy (Version 4.113.1, by Specs GmbH, Berlin, Germany). The pass energy was set at 20 eV, giving a full width at half maximum (FWHM) of 0.9 eV for the Ag 3d_5/2_ peak. To analyze the C 1s and O 1s data, Gaussian/Lorentzian convolution functions were employed with a Shirley background to fit the spectra by the CasaXPS software (Version 2.3.15). Concerning the C 1s spectrum, the primary component, which is situated at a binding energy (BE) of 284.4 eV, exhibits an asymmetric shape that tends toward higher binding energies [41]. The line asymmetry of this component was characterized using the Doniach–Šunjić (DS) function with an asymmetry coefficient of a = 0.05 according to literature [42]. All other components in the photoelectron spectra exhibit symmetry. The FWHM was about 2.3 eV for O 1s and about 1 eV for the main graphitic peak of C 1s, with a standard deviation of about ±0.1 eV for the binding energy position of the peaks.

Transmission electron microscopy (TEM) images were captured using a JEOL 2100 microscope (200 kV). Thermogravimetric analysis (TGA) was performed using a TGA55 (TA Instruments, New Castle, DE, USA) under an inert nitrogen atmosphere. The temperature was increased to 90 °C at a ramp rate of 10 °C/min and remained isothermal until a constant weight was reached, followed by a linear increase to 800 °C at a ramp rate of 5 °C/min. The specific surface area and total pore volume of MWCNTs were analyzed using an Autosorb IQ TPX by Quantachrome (Boynton Beach, FL, USA), and calculations were performed using the Brunauer–Emmett–Teller (BET) method [43].

Zeta potentials were measured using a Malvern Panalytical Zetasizer-NanoZS at 25 °C (Malvern, UK). For these measurements, NaCl was used to disperse the MWCNTs, and three measurement rounds were conducted with no intermediate pauses. Initially, various aqueous solutions with different initial pH values (ranging from 2.0 to 10.0) were prepared by adjusting the pH with 0.1 M HCl or 0.1 M NaOH [44]. Subsequently, 2 mg of MWCNTs were added to 50 mL of each aqueous solution, and the mixtures were sonicated in a sonication bath (S30 Elmasonic, 50 kHz, 75 W, Singen, Germany) for 15 min to ensure proper dispersion before performing the measurements.

### 2.5. Batch Adsorption Study

Adsorption of MB by pristine or oxidized MWCNTs by dispersing the nanotubes in a water solution containing a specific amount of MB (m_MWCNTs_:10.5 mg, volume: 15 mL, pH: ~6). The mixture was placed in tightly sealed glass bottles and agitated on a rotor at 12 rpm and a constant temperature of 28 °C within an incubator (Witeg, GmbH, Wertheim, Germany). A parametric study was performed to investigate the effect of contact time between the adsorbent and MB, as well as the initial MB concentration ranging from 10 to 150 mg/L, on the adsorption capacity.

At the end of each experiment, samples were collected and centrifuged for 10 min at 9000 rpm. The supernatant was then analyzed using a UV–Vis spectrophotometer (Shimadzu, UV-1900, Kyoto, Japan). For UV/Vis analysis, the characteristic absorption band of MB at 663 nm was monitored to quantify its concentration in the solution [45]. Each data point in the adsorption experiment results presented herein represents the average value of at least three measurements.

The MB removal efficiency (R), the amount of MB adsorbed at time t ($q_{t}$, mg/g), and the amount of MB adsorbed at equilibrium ($q_{e}$, mg/g) were calculated using the following equations:

(1) $$R\left(\%\right)=\frac{Co-Ce}{Co}\;\times \;100$$

(2) $$q_{t}=\frac{Co-Ct}{m}\;\times \;V$$

(3) $$q_{e}=\frac{Co-Ce}{m}\;\times \;V$$

where $C_{0}$, $C_{e}$, and $C_{t}$ represent the initial, equilibrium, and specific time (t) concentrations of MB (mg/L), respectively. V (L) denotes the volume of the solution and m is the adsorbent’s mass (g).

## 3. Results and Discussion

### 3.1. Effect of Treatment Time and Pulse Voltage on the Oxidation Degree of MWCNTs

The experimental conditions for plasma-oxidized nanotubes, including treatment time and pulse voltage, are listed in Table 1. To confirm successful modification, surface elemental analysis via XPS took place for all samples. The only elements present in the samples were oxygen and carbon (Figure S1). The O/C atom ratio derived from the corresponding C 1s and O 1s XPS spectra are also presented in Table 1, along with those of pristine MWCNTs and chemically modified MWCNTs (Chem). The O/C ratio is a direct indicator of the percentage of oxygen-bearing groups introduced onto the nanotube walls.

The XPS results confirm that the changes in the MWCNT surface chemistry were closely related to the plasma conditions. Generally, plasma treatments resulted in an exponential increase in the O/C ratio with treatment time (Table 1). The degree of oxidation increased with increasing treatment time and applied pulse voltage. Oxidation within just the first 10 min of nanopulsed-DBD plasma treatment at 31 kV (Pl 31_10 sample) succeeded to a higher degree (O/C: 0.063) than with chemical treatment (O/C: 0.051). After 60 min of plasma treatment (Pl 31_60 sample), the O/C ratio reached a value 4.5 times higher (O/C: 0.090) compared to pristine nanotubes and ~76% higher than that of chemically oxidized nanotubes.

### 3.2. Energy Efficiency of the Nanopulsed-DBD

After 10 min of plasma treatment, the O/C atom ratio reached the value of 0.063, corresponding to an atomic oxygen surface area of ~6 at.%. The latter increased to ~8.3 at.% after 60 min of treatment (Table 1). Considering the mass of MWCNTs modified in each experiment (50 mg) and the discharge power (~0.95 W), the energy efficiency ranges from ~50–300 g_MWCNTs_/kWh for treatment times spanning 10 to 60 min. This demonstrates the exceptionally low operational cost of the present nanopulsed-DBD system. A comparison of the oxidation efficiency (measured by the increase in O/C atom ratio) under the conditions of this study with experimental data from other research studies on nanotube modification using non-thermal plasma was conducted (Table 2). Notably, the power consumed in this work (~1 W) is significantly lower than that of other studies, which can reach up to 700 W while achieving a similar increase in the oxidation degree (~350%) [46]. The high degree of oxidation of nanotubes combined with minimal power consumption suggests that the present DBD reactor, energized by high-voltage nanopulses, has strong potential as a highly cost-effective technology for modifying nanotubes and introducing oxygen functional groups onto their surface.

### 3.3. Physicochemical and Morphological Characterization

#### 3.3.1. XPS Analysis

**Figure 2:**
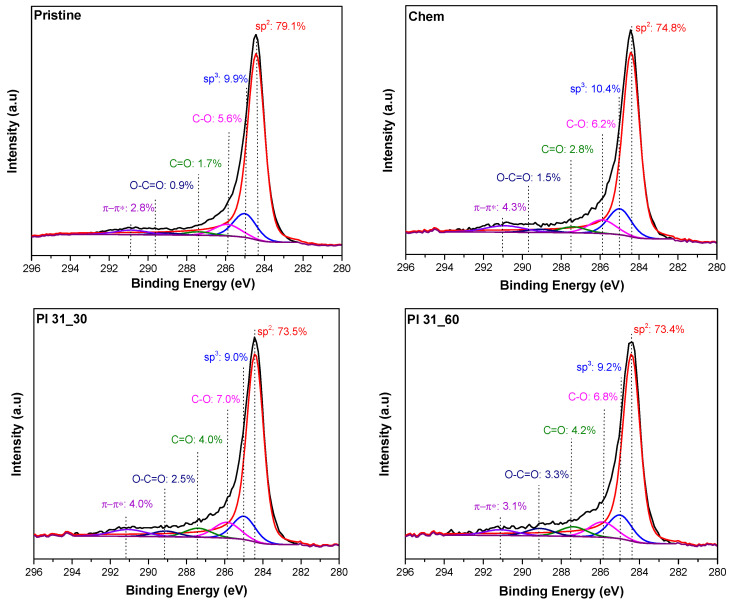
C 1s deconvoluted XPS spectra of pristine, chemically oxidized, and two representative plasma-oxidized MWCNTs samples.

Representative examples of the obtained C 1s spectra of the plasma-treated MWCNTs are shown in Figure 2, along with the corresponding spectra of pristine and chemically functionalized MWCNT samples for comparison. The deconvolution of the spectra and the respective peak assignment, consistent with recent literature [54,55,56,57], are included in Figure 2. In addition to the main peak representing the graphitic structure at 284.4 eV (sp^2^ C-C), peaks of interest include those at 285 eV (assigned to sp^3^ carbon) and at 285.9, 287.4, and 289.1 eV assigned to various types of carbon–oxygen groups. The relative percentage of each peak is also shown in Figure 2. The main graphitic feature remained dominant after the different treatments, although its percentage in the sample decreased with increasing plasma treatment time, indicating that reaction and rehybridization occurred, giving rise to other peaks. Along with the increase in the O/C ratio, these XPS results confirm the successful modification.

**Figure 3:**
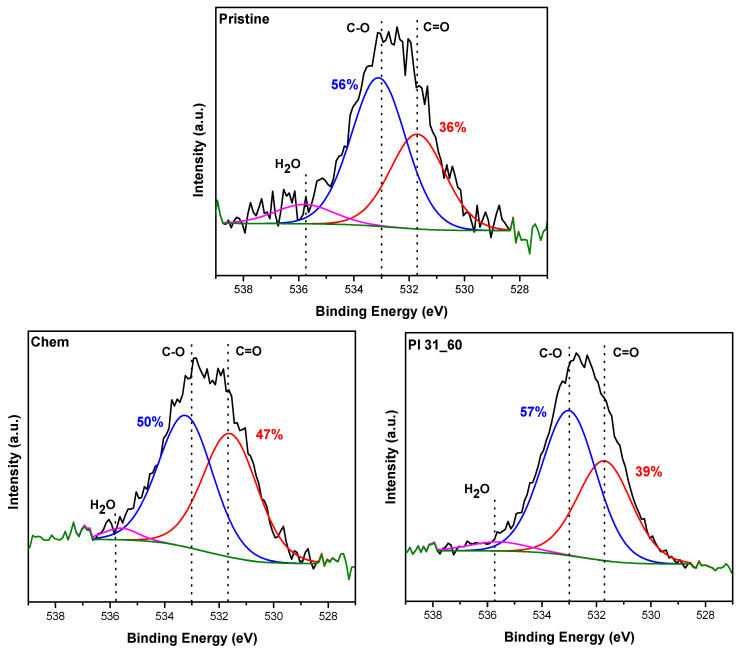
O 1s deconvoluted XPS spectra of pristine, chemically oxidized, and plasma-oxidized MWCNTs.

To better clarify the chemistry and the introduced oxygen groups on the surface of the nanotubes, analysis was performed through the deconvolution of the O 1s XPS spectra. Indicative XPS spectra are shown in Figure 3, where the peak analysis is also illustrated. For this analysis, two major carbon–oxygen groups were considered [26,58]. The first category with an XPS peak at about 533 eV pertains to the C-O bond, which includes contributions from hydroxyl/phenol (-C-OH), ether (-C-O-C-), as well as the underlined oxygen on ester moieties (C-O-C=O). The second peak at 531.6–531.7 eV corresponds to groups with C=O double bonds, such as carboxyl (O=C-OH), ketone (-C=O), and carbonyl (O-C=O) moieties. The difference in the BE of the contributing groups of the second peak is small [59,60], and thus in this study we treat them as one family of moieties. Based on the XPS spectra, the percentage of the C-O single bond groups was clearly higher in all samples processed with plasma. In contrast, chemically oxidized nanotubes showed a balanced percentage between the two types of oxygen groups.

**Figure 4:**
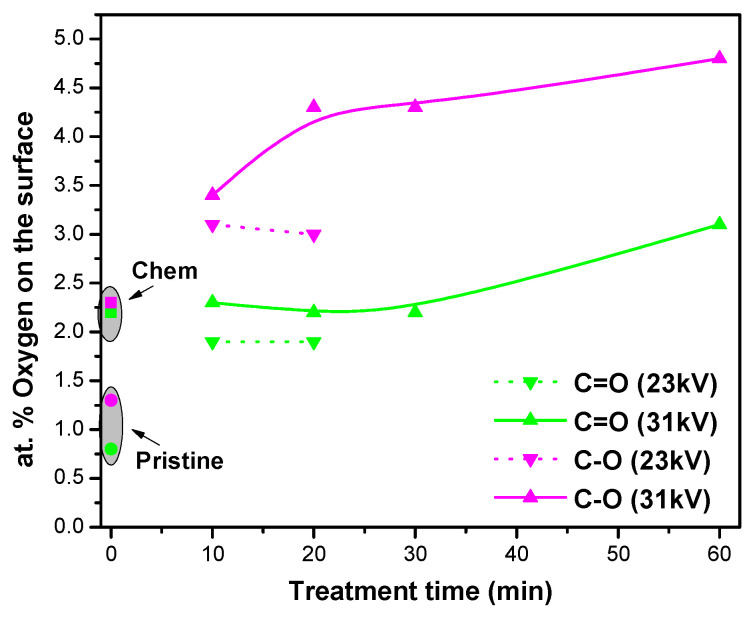
Percentage of surface oxygen atoms of the two main oxygen-containing groups, as derived from the deconvolution of the corresponding O 1s XPS spectra. Samples were treated with plasma at 23 kV (dotted lines) or 31 kV (solid lines). The points at time 0 min correspond to the respective percentages of pristine or chemically treated MWCNTs.

Figure 4 presents the surface percentage (at.%) of the two main oxygen groups, C-O and C=O, obtained for each applied pulse voltage as a function of treatment time. For comparison, the corresponding percentages of pristine and chemically modified MWCNTs are also shown in Figure 4 (at 0 min). As can be observed, plasma modification at the lowest voltage (23 kV) introduced oxygen groups with twice the C=O and three times the C-O percentages compared to pristine MWCNTs. The materials treated with plasma at the highest pulse voltage (31 kV) had similar percentages of C=O groups with chemically modified MWCNTs. Up to 30 min of plasma treatment, the C=O content remained constant, while the C-O content increased with prolonging time from 10 to 60 min of treatment. After 60 min of plasma treatment, the oxygen group content significantly increased, reaching 4.8 at.% and 3.1 at.% for the C-O and C=O groups, respectively. Interestingly, under these nanopulsed-plasma conditions, the attached C-O groups did not further react over time to increase the presence of C=O groups, as often reported in the literature [47,61,62]. Instead, C-O groups predominate over carbonyl moieties and analogs. The formation of ether groups is very likely, while hydroxyl groups can be formed upon interaction with hydrogen atoms from neighboring atoms or through interaction with atmospheric moisture when the nanotube surface is exposed to the atmosphere after plasma treatment [62].

#### 3.3.2. TEM Analysis

**Figure 5:**
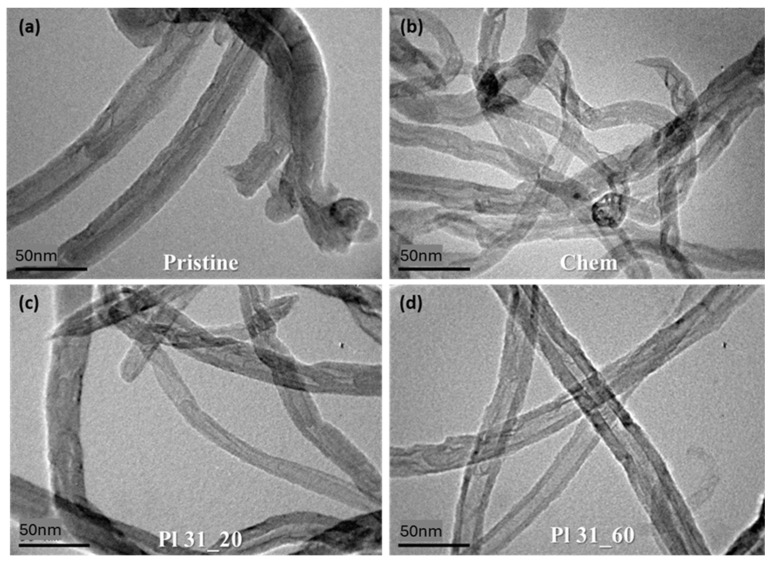
Selected TEM images of (**a**) pristine MWCNTs, (**b**) chemically oxidized MWCNTs, (**c**) Pl 31_20, and (**d**) Pl 31_60.

TEM images were obtained to evaluate changes in the surface morphology of MWCNTs after both chemical and plasma treatment. Representative examples are presented in Figure 5. The images show that plasma-modified MWCNTs after 20 and 60 min of treatment did not exhibit significant structural alterations compared to the pristine nanotubes. This observation aligns with previous studies, which also did not note substantial structural differences in MWCNTs before and after plasma treatment [47], indicating that nanopulsed-DBD plasma modification is not destructive to the overall bulk structure of the nanotubes. However, the presence of reasonable defects at various points in the nanotube structure was noticeable, and these defects appeared to increase with longer plasma treatment times. Similar defects were observed in chemically oxidized MWCNTs.

#### 3.3.3. BET Analysis

**Figure 6:**
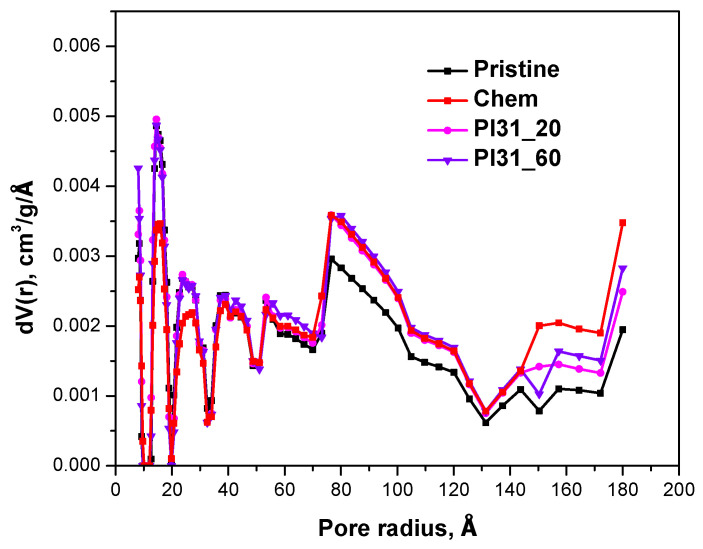
Pore size distributions of pristine, chemically modified, and plasma-modified (Pl31_20 and Pl 31_60) MWCNTs.

The BET surface analysis results indicated no significant changes in the specific surface area (Table 3), with the pristine and modified MWCNT samples exhibiting surface areas ranging between 111 and 96 m^2^/g. The total pore volume of the samples was determined using the DFT method. For the pristine and chemically modified nanotubes, the total pore volumes were 0.111 cm^3^/g and 0.119 cm^3^/g, respectively. However, a significant change was observed in the plasma-modified nanotubes, with the total pore volume increasing to 0.306 cm^3^/g. Figure 6 shows the pore size distributions for the nanotube samples. The pore size (half pore width) of pristine and chemically oxidized MWCNTs ranged from ~10 to 50 Å, while that of Pl 31_60 ranged from 10 to 180 Å.

#### 3.3.4. TGA

**Figure 7:**
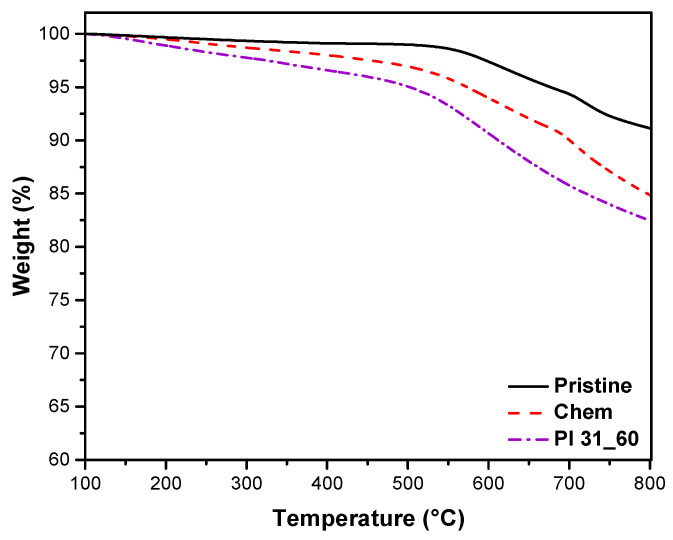
Thermogravimetric analysis under nitrogen atmosphere of pristine, chemically treated, and plasma-treated MWCNTs.

Figure 7 shows the thermogravimetric curves of pristine, chemically modified, and plasma-modified nanotubes under an inert atmosphere. Thermal degradation of pristine nanotubes was observed at a temperature of 600–700 °C, likely starting from defects in the nanotube walls. Conversely, nanotubes with oxygen groups on their surface, whether chemically or plasma-treated, exhibited weight loss starting at lower temperatures (~150–200 °C) due to the decomposition of the oxygen groups in their structure. The modification introduced further structural defects, making the nanotubes more susceptible to thermal degradation. Pl 31_60 showed a higher weight loss than that of Chem, reflecting its higher oxidation degree. At 800 °C, pristine, chemically treated, and plasma-treated MWCNTs retained 91%, 85%, and 83% of their original weight, respectively.

#### 3.3.5. Measurement of Zeta Potential

**Figure 8:**
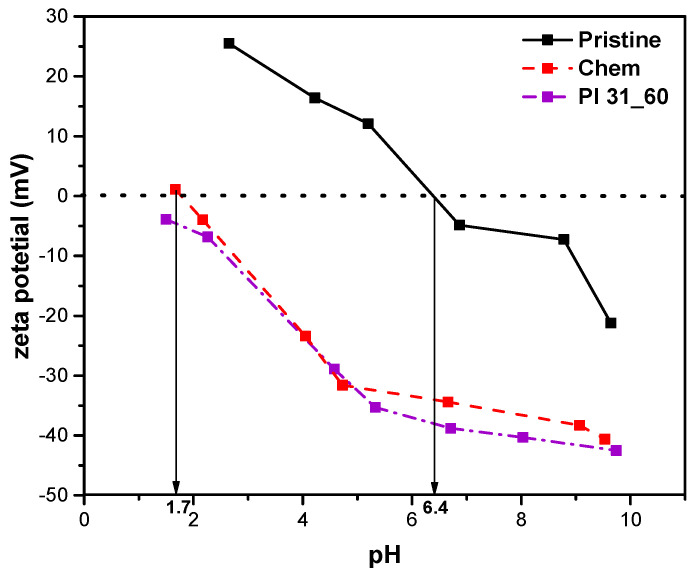
Effect of pH on zeta potential of pristine, chemically treated, and plasma-treated nanotubes.

Figure 8 illustrates the determination of z-potential versus pH for the three types of nanotubes. As shown, the pH_PZC_ for pristine MWCNTs was 6.4, for chemically oxidized it was 1.7, while plasma-oxidized nanotubes (Pl 31_60) did not present a pH_PZC_ within the studied range due to their highly negative surface charge, indicating that plasma modification introduced a higher percentage of oxygen groups. When nanotubes are placed in a solution with a pH lower than their pH_PZC_, their surface becomes positively charged, repelling positively charged particles present in the solution. Conversely, if the solution’s pH is higher than the pH_PZC_, the nanotube’s surface becomes negatively charged, attracting positively charged ions. Therefore, in solutions with a pH value higher than the pH_PZC,_ the adsorption of MB cationic dye on the nanotubes is expected to be favored. In this study, where the solution pH was ~ 6, the surface of the oxidized nanotubes is expected to be negatively charged, leading to the attraction of the positively charged MB ions.

### 3.4. Adsorption of MB onto Pristine, Chemically Oxidized, and Plasma-Oxidized MWCNTs

#### 3.4.1. Effect of Adsorption Time

**Figure 9:**
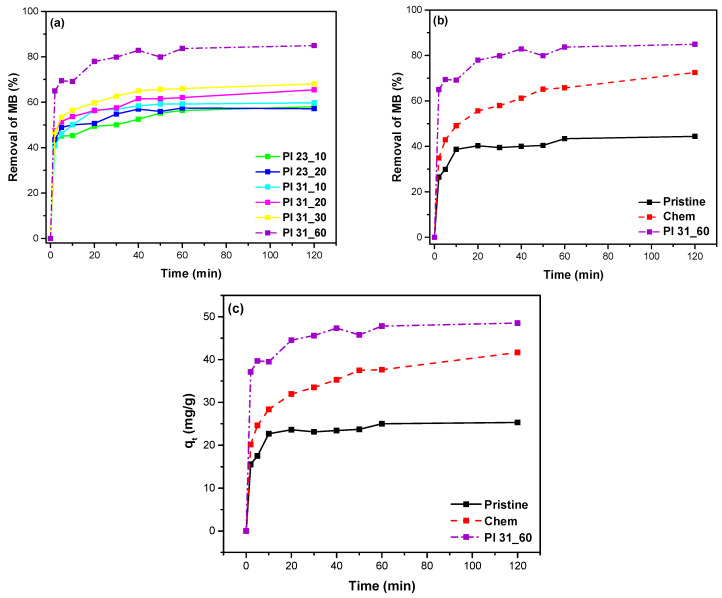
Removal efficiency of MB through adsorption as a function of adsorption time for (**a**) various plasma-treated samples and (**b**) pristine, chemically oxidized, and plasma-oxidized (Pl 31_60) MWCNTs. (**c**) Adsorption capacity (*q_t_*) of pristine, chemically, and Pl 31_60 MWCNTs as a function of time (*t*) (initial MB concentration: 40 mg/L).

The removal efficiency of MB through adsorption onto the plasma-modified samples at different treatment times and pulse voltages is presented in Figure 9a. All plasma-modified samples exhibited increased MB adsorption compared to pristine samples, with a more significant increase as the plasma modification time extended. Even for the sample treated with plasma for 10 min at the low pulse voltage of 23 kV (Pl 23_10), the removal efficiency of MB reached 58%, surpassing the efficiency of pristine MWCNTs (45%). Notably, the plasma-oxidized sample for 60 min (Pl 31_60) showed the highest MB adsorption (~85%), outperforming both all other plasma-treated samples and the chemically modified sample.

Considering its superior efficiency towards MB removal from water, the sample treated for 60 min was chosen as the adsorbent for further study and comparison with pristine and chemically oxidized MWCNTs, as presented in Figure 9b. After 2 h of contact time, the MB removal was 44.4% for pristine, 72.5% for chemically oxidized, and 84.9% for Pl 31_60 MWCNTs. Additionally, the effect of contact time on the adsorption capacity ($q_{t}$) for MB of these three types of MWCNTs (pristine, chemically oxidized, and plasma-oxidized Pl 31_60) is shown in Figure 9c. It is noteworthy that the Pl 31_60 managed to remove MB in a very high percentage, as this was observed from the first 20 min and seems to show partial saturation up to 2 h. After 2 h of contact time, the adsorption capacities were 25.18 mg/g for pristine, 41.60 mg/g for chemically oxidized, and 48.58 mg/g for plasma-oxidized Pl 31_60 MWCNTs.

**Figure 10:**
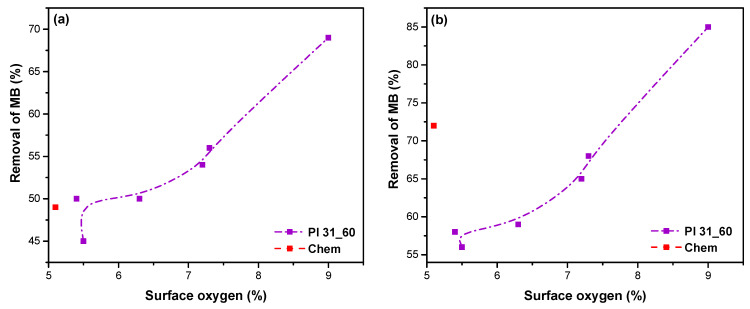
Removal efficiency of MB as a function of the percentage of surface oxygen in the plasma-modified samples for adsorption time: (**a**) 10 min and (**b**) 2 h. In all cases, the initial MB concentration was 40 mg/L. The corresponding values of Chem. MWCNTs are also presented.

Figure 10 shows the MB removal after (a) 10 min and (b) 2 h of adsorption as a function of the content of oxygen on the surface of the nanotubes, as derived from XPS, corresponding to the different plasma modification treatment durations. These plots demonstrate that increasing the oxidation degree of the nanotubes linearly enhanced the removal of MB from water. This trend confirms that longer plasma modification times gradually introduced more negatively charged oxygen functional groups (-COOH and -OH) on the surface of the nanotubes, resulting in stronger attractive interactions between the negatively charged nanotube surface and the cationic dye molecules. A comparison with chemically modified nanotubes is also provided. In the case of 10 min of adsorption, the removal of MB using the chemically modified samples was ~49% (Figure 10a). This removal percentage is similar to Pl 23_20, which also has a comparable percentage of oxygen groups, Table 1. Interestingly, after 2 h of adsorption, chemically oxidized MWCNTs exhibited a relatively high removal of ~72% despite having a lower oxygen percentage (~5 at.%) (Figure 10b). This higher removal percentage may be attributed to different surface chemistry that cannot be distinguished by the broad classification of Figure 2 (e.g., different types of C-O groups) or to other, kinetically slower adsorption mechanisms.

#### 3.4.2. Effect of Pollutant Concentration

**Figure 11:**
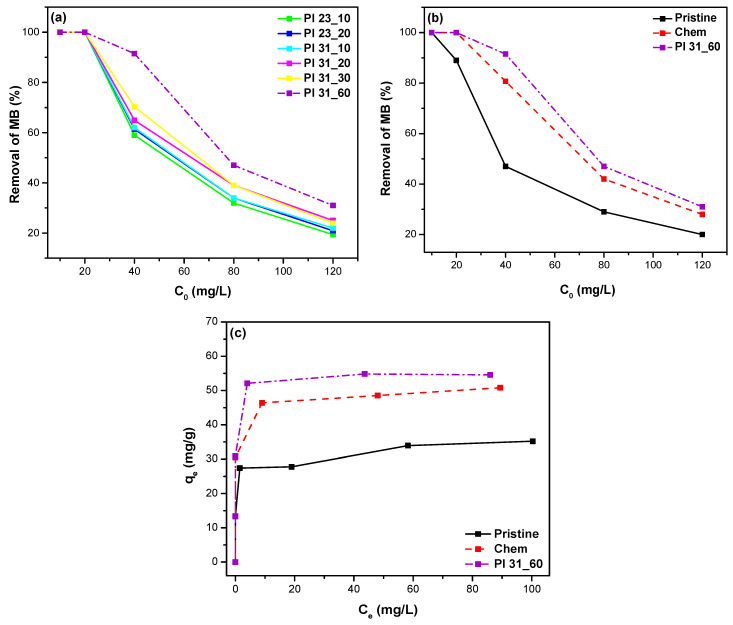
Removal efficiency of MB through adsorption as a function of initial pollutant concentration for (**a**) various plasma-treated samples and (**b**) pristine, chemically oxidized, and plasma-oxidized (Pl 31_60) MWCNTs. (**c**) The amount of MB adsorbed at equilibrium (*q_e_*) onto pristine, chemically and Pl 31_60 MWCNTs (adsorption time: 24 h).

To determine the removal efficiency and the adsorption capacity at equilibrium, *q_e,exp_*, the dye solutions were kept in contact with the adsorbents for 24 h. The performance of several plasma-oxidized samples in adsorbing and removing MB from water was tested (Figure 11a). As shown, the plasma-treated sample that resulted in the highest MB removal, surpassing even Chem. nanotubes, was the Pl 31_60.

Figure 11b presents a direct comparison of pristine, chemically oxidized, and plasma-oxidized Pl 31_60 MWCNTs at different initial MB concentrations. At an initial MB concentration of 40 mg/L, the removal efficiencies were 47%, 81%, and 92% for pristine, chemically modified, and plasma-modified MWCNTs, respectively, whereas at the maximum initial concentration of 120 mg/L, the removal efficiencies for the same MWCNTs were 20%, 28%, and 31%, respectively. Figure 11c shows the adsorbed amount of MB per unit mass of adsorbent (adsorption capacity), $q_{e}$ (mg/g), for the different samples. The maximum adsorption capacity was that of plasma-modified nanotubes Pl 31_60 (~55 mg/g), followed by Chem (~50 mg/g), and finally the pristine nanotubes (~35 mg/g).

### 3.5. Adsorption Kinetics and Isotherm Models

**Figure 12:**
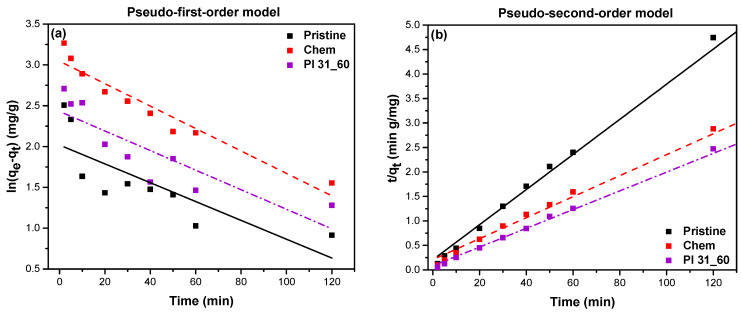
Plots of (**a**) pseudo-first-order model and (**b**) pseudo-second-order model for pristine, chemically oxidized, and plasma-oxidized (Pl 31_60) MWCNTs.

Pseudo-first-order and pseudo-second-order models were applied to investigate the kinetics of MB adsorption on pristine, chemically oxidized, and plasma-oxidized Pl 31_60 MWCNTs, using Equations (4) and (5), respectively.

(4) $$\ln\left(q_{e}-q_{t}\right)=lnq_{e}-k_{1}t\;$$

(5) $$\frac{t}{q_{t}\;}=\frac{1}{k_{2}q_{e}^{2}}+\frac{t}{q_{e}}\;$$

where $k_{1}$ (1/min) and $k_{2}$ (g/mg min) are rate constants for the different models [63]. According to the pseudo-first-order kinetic model, the rate of adsorption is proportional to the available sites on the adsorbent, commonly the case of the physisorption type of adsorption driven by weak van der Waals forces. For this model, $ln(q_{e}-q_{t})$ was plotted as a function of t, and $k_{1}$ and $q_{e}$ were calculated from the slope and intercept, respectively (Figure 12a). For the pseudo-second-order model, tqt was plotted as a function of t, and $k_{2}$ and $q_{e}$ were obtained from the slope and intercept, respectively (Figure 12b). The parameters for the pseudo-first-order and pseudo-second-order models are listed in Table 4. The correlation coefficients ($R^{2}$) for the pseudo-first-order model ranged from 0.647 to 0.930 for all adsorbent materials, and the predicted adsorption capacities ($q_{e,cal}$) were less than the experimental values ($q_{e,exp}$) (Table 4). Therefore, the kinetics of adsorption cannot be satisfactorily described by this model. In contrast, the second-order model fitted the experimental data perfectly ($R^{2}$ > 0.99) for the entire duration of the adsorption process. It also satisfactorily predicted the adsorption capacity of all types of nanotubes, as the $q_{e,cal}$ values were comparable to the $q_{e,exp}$ values (Table 4).

**Figure 13:**
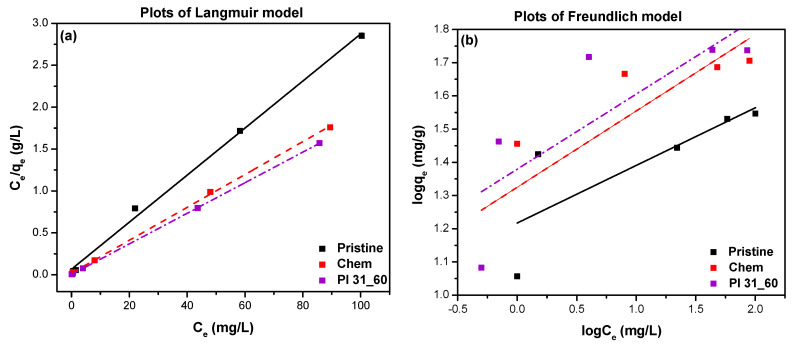
Plots of (**a**) Langmuir model and (**b**) Freundlich model for pristine, chemically oxidized, and plasma-oxidized (Pl 31_60) MWCNTs.

The data of MB adsorption were also fitted to the Langmuir and Freundlich isotherm models to elucidate the underlying adsorption mechanisms [64]. The Langmuir model posits that adsorption occurs on a homogeneous surface with energetically equivalent sites without lateral interaction between adsorbed molecules, assuming a monolayer adsorption motif [5,65]. Conversely, the Freundlich isotherm model describes adsorption on a heterogeneous surface with varying adsorption energies, implying that adsorption capacity increases exponentially with increasing concentration, reflecting multilayer sorption [66,67]. The linearized forms of the Langmuir and Freundlich equations are expressed as follows:

(6) $$\frac{C_{e}}{q_{e}}=\frac{1}{Q_{b\;}}+\frac{C_{e}}{Q}$$

(7) $$logq_{e}=logK+\frac{1}{n\;}logC_{e}$$

where $q_{e}$(mg/g) is the amount of pollutant adsorbed per unit mass of adsorbent at equilibrium; Q (mg/g) is the maximum adsorption capacity of the adsorbent corresponding to monolayer coverage; and b (L/mg) is the Langmuir adsorption constant, which determines the affinities of binding sites and the sorption free energy. Moreover, $C_{e}$ (mg/L) is the equilibrium concentration of the pollutant in solution; K ((mg/g) (L/mg)^1/n^) is the Freundlich adsorption constant related to the maximum adsorption capacity of the adsorbent; and $\frac{1}{n}$ is a constant related to the adsorption intensity, varying with the heterogeneity of the adsorbent. The dimensionless constant separation factor, *R_L_*, is an indicator of the adsorption capacity, and it is calculated as follows:

(8) $$R_{L}=\frac{1}{1+bC_{0}}$$

where *C_0_* (mg/L) is the initial pollutant concentration in aqueous solution. The adsorption process is considered favorable when 0 < $R_{L}$ < 1 and unfavorable when $R_{L}$ > 1. For the Langmuir model, Ceqe was plotted as a function of $C_{e}$, and Q and b were calculated from the slope and intercept, respectively (Figure 13a). For the Freundlich model, (${logq}_{e}$) was plotted as a function of (${logC}_{e}$), and n and K were calculated from the slope and intercept, respectively (Figure 13b). The calculated Langmuir and Freundlich isotherm constants, along with the correlation coefficients ($R^{2}$) are presented in Table 5. Based on the $R^{2}$ values, the Langmuir model accurately described the adsorption of MB on the three types of nanotubes, as shown in Figure 13a. The maximum adsorption capacities were determined to be 35.52, 50.60, and 54.67 mg/g for pristine, chemically modified, and plasma-modified MWCNTs, respectively. The $R_{L}$ and $\frac{1}{n}$ values indicated the favorable nature of the adsorption in both cases (0 < $R_{L}$ < 1 and $\frac{1}{n}$ < 1), signifying strong adsorption interactions between the adsorbent and the adsorbate. Similar results have been reported by Zhao et al. (2022) [47] and Konicki and Pelech (2018) [68], who found that the Langmuir isotherm more satisfactorily described the adsorption of MB or other cationic pigments on MWCNTs compared to the Freundlich isotherm.

### 3.6. Analysis of the Adsorption Mechanisms

MB adsorption, as inferred from material characterization, adsorption isotherms, and kinetic experiments, is best described by the monolayer adsorption and the pseudo-second-order kinetics models. Certainly, for all the materials of this study, π-π interactions between the aromatic rings of MB and the aromatic rings of MWCNTs contribute to the adsorption of MB [69]. Specifically, for oxidized samples, a substantial amount of oxygen-bearing groups was noted on the surface of the modified nanotubes, as confirmed by XPS analysis. The ratio and nature of functional groups in each adsorbent material significantly affect its adsorption capacity. Indeed, the removal of MB increased linearly with their content in the plasma-treated nanotubes (Figure 10). Furthermore, the adsorptive capacity is highly dependent on pH. Therefore, the point of zero charge (pH_PZC_) was determined through the z-potential of the adsorbent materials. The adsorption of a cationic dye such as MB is favored at pH > pH_PZC_ due to the presence of functional groups such as -OH and -COOH on the surface of the nanotubes, which make it negatively charged. In the case of the chemically oxidized and plasma-oxidized nanotubes of this work (both with pH_PZC_ < 2), electrostatic interactions were developed between the positively charged MB and the negatively charged groups. Thus, by evaluating the results, it is evident that the primary mechanism of adsorption is driven by electrostatic interactions in the case of oxidized MWCNTs. Another possible mechanism involves the hydrogen bonds that can be formed between the nitrogen (-N) atoms in the MB structure and the functional groups (e.g., -OH) on the surface of MWCNTs.

### 3.7. Comparison of the Adsorption Capacity with Other Studies

A comparison between the maximum adsorption capacity, Q, of pristine and modified nanotubes in the present work and other research studies focusing on cationic dyes, particularly MB, was conducted and is presented in Table 6. In this study, the adsorption capacity of pristine MWCNTs increased by ~54% after 60 min of plasma treatment (from ~35.5 to ~54.7 mg/g), whereas in another study a 17% increase was reported after plasma modification [47]. Additionally, nanotubes modified with strong chemicals showed high adsorption capacities, such as 188.7 mg/g for MWCNT-COOH and 319.1 mg/g for P-CSCNT [11]. However, strong oxidizing agents can damage their structure and require significant energy consumption. Some studies reported comparable or even lower adsorption capacities of modified MWCNTs [70,71] compared to the modified nanotubes of the present study. Carbon nanotubes generally exhibit lower adsorption capacities compared to porous materials like activated carbon (AC), graphene oxide (GO), and bentonite due to their smaller specific surface area [72,73].

## 4. Conclusions

In this study, MWCNTs were oxidized using cold plasma for subsequent use as adsorbents for the removal of the cationic dye Methylene Blue (MB) from water. For this purpose, a dielectric barrier discharge (DBD) reactor at atmospheric pressure, driven by high-voltage nanosecond pulses, was used.

XPS, TEM, BET, and TGA confirmed the introduction of oxygen groups, which impart a negative charge onto the surface of the plasma-modified nanotubes without notably altering their structure. Compared to chemically modified nanotubes, which showed a balanced ratio of C-O (alcohol, ester, and ether) and C=O carbonyl groups, plasma-oxidized nanotubes exhibited increased C-O groups’ content. Increased discharge power and longer treatment times resulted in higher oxidation degrees of the nanotubes. Maximum plasma modification (O/C = 0.09 from O/C = 0.02 for pristine MWCNTs and O/C = 0.05 for chemically oxidized) was achieved at a pulse frequency of 200 Hz and a pulse voltage of 31 kV after 60 min of treatment. Depending on the plasma treatment time, the energy efficiency of the current system was found to range from ~50 to 300 g_MWCNTs_/kWh, demonstrating the low operational cost of DBD modification when combined with voltage nanosecond pulses.

It was demonstrated that the plasma oxidation time of the nanotubes gradually increased their oxidation degree and, consequently, the MB removal from water. The plasma modification for 60 min at 31 kV showed the highest removal efficiency of MB from water. This plasma-treated sample exhibited a high removal of MB from water (~65%) after just 2 min of adsorption, while the pristine and chemically oxidized MWCNTs achieved lower removal efficiencies (~27 and 40%) in the same time period. After 2 h of contact, the plasma-treated sample removed ~85% of MB from water. The experimental adsorption data fitted more satisfactorily to the pseudo-second-order kinetic model and the Langmuir isotherm model. Regarding the adsorption capacities of the nanotubes for MB, they were 35.52, 50.60, and 54.67 mg/g for the pristine, chemically oxidized, and plasma-oxidized, respectively, indicating a significant increase (~54%) in the adsorption capacity of the nanotubes after plasma treatment. Therefore, plasma-modified nanotubes could be effectively used for the removal of MB dye from water. This work provides motivation for further study and utilization of plasma as a low-cost, environmentally friendly technology for material modification and water purification applications.

## Figures and Tables

**Table 1:** O/C atom ratio of pristine, chemically oxidized, and plasma-oxidized MWCNTs at different processing times and pulse voltages (23 and 31 kV).

Sample	Treatment Time (min)	Pulse Voltage (kV)	O/C
Pristine	-	-	0.020
Chem	-	-	0.051
Pl 23_10	10	23	0.055
Pl 31_10	10	31	0.063
Pl 23_20	20	23	0.054
Pl 31_20	20	31	0.072
Pl 31_30	30	31	0.073
Pl 31_60	60	31	0.090

**Table 2:** Comparative analysis of the O/C ratio determined from various research studies on nanotube modification using plasma technology.

Plasma System	Gas	Treatment Time (min)	Initial O/C	Final O/C	Power (W)	Ref.
Nanopulsed Dielectric Barrier Discharge	Air	10	0.020	0.063	~0.95	Present study
Nanopulsed Dielectric Barrier Discharge	Air	60	0.020	0.090	~0.95	Present study
Microwave-Excited Surface Wave)	Ar/O_2_	15	0.127	0.701	700	[46]
Microwave-Excited Surface Wave	O_2_	15	0.127	0.509	700	[46]
Radio Frequencies	O_2_	30	0.009	0.13	50	[37]
Dielectric Barrier Discharge	O_2_	30	0.011	0.025	-	[47]
Corona Discharge	Air	30	0.010	0.038	21	[48]
High Frequency Generator	O_2_/Ar	5	0.017	0.148	80	[49]
Microwave-Excited Surface Wave	Ar/Ar-H_2_O	Ar (5 min)/ Ar-H_2_O (10 min)	0.127	0.449	700	[50]
Dielectric Barrier Discharge	O_2_	40	0.019	0.081	15	[51]
Radio Frequencies	O_2_	10	0.013	0.103	-	[52]
Radio Frequencies	Air	10	0.013	0.063	-	[52]
Radio Frequencies	Ar-O_2_	60	0.031	0.138	40	[53]

**Table 3:** BET-specific surface area and pore volume of pristine and modified MWCNTs.

Sample	Surface Area BET (m^2^/g)	Pore Volume (cm^3^/g)
Pristine	107	0.302
Chem	116	0.340
Pl 31_20	118	0.345
Pl 31_60	124	0.359

**Table 4:** Parameters of the kinetic models for MB adsorption on pristine, chemically oxidized, and plasma-oxidized (Pl 31_60) MWCNTs.

Adsorbent	Pseudo-First-Order Model				Pseudo-Second-Order Model		
	$q_{e,exp}$ (mg/g)	$k_{1}$ (min)	$q_{e,cal}$ (mg/g)	$R^{2}$	$k_{2}$ (g/mg min)	$q_{e,cal}$ (mg/g)	$R^{2}$
Pristine	27.79	0.011	7.52	0.647	0.006	27.89	0.999
Chem	46.37	0.014	21.00	0.930	0.002	46.66	0.999
Pl 31_60	52.13	0.012	11.36	0.749	0.004	52.27	0.999

**Table 5:** Parameters of Langmuir and Freundlich models for MB adsorption on pristine, chemically oxidized, and plasma-oxidized (Pl 31_60) MWCNTs.

Adsorbent	Langmuir Model					Freundlich Model			
	$q_{e,exp}$ (mg/g)	Q (mg/g)	b (L/mg)	$R_{L}$	$R^{2}$	n	K (mg/g) (L/mg) 1/*n*	$\frac{1}{n}$	$R^{2}$
Pristine	35.20	35.52	0.001	0.97–0.76	0.996	5.76	16.490	0.173	0.638
Chem	50.82	50.60	0.000	0.99–0.92	0.999	4.35	21.135	0.229	0.748
Pl 31_60	54.60	54.67	0.000	0.99–0.95	0.999	4.42	23.962	0.225	0.651

**Table 6:** Comparison of the maximum adsorption capacity between various nanotubes.

Adsorbent	Treatment Oxidation	Dye	pH	Concentration of Dye (mg/L)	Adsorption Capacity, Q (mg/g)	Adsorption Time (h)	Reference
Pristine	-	MB	~6	120	35.52	24	This study
Chem	chemical	MB	~6	120	50.82	24	This study
Pl 31_60	plasma	MB	~6	120	54.60	24	This study
CNT0	-	MR	6	35	68.44	1	[47]
CNT60	plasma	MR	6	35	80.33	1	[47]
MWCNT-COOH	chemical	MR	4	20	108.69	0.42	[23]
AC	chemical	MB	6	100	270.27	5	[72]
GO	chemical	MB	6	100	243.90	5	[72]
CNTs	chemical	MB	6	100	188.68	5	[72]
Magnetite -MWCNTs	chemical	MB	6.5	30	48.06	2	[70]
CNTs	chemical	MB	7	20	46.20	1	[71]
Plasma-treated bentonite	plasma	MB	-	200	303	-	[73]
MWCNTs	-	MB	7	1.4–37.4	15.87	24	[74]
MMWCNTs	magnetic	MB	7	1.4–37.4	15.74	24	[74]
CSCNT	-	MB	6	25	50.5	3	[11]
P-CSCNT	chemical	MB	6	150	319.1	3	[11]

## Data Availability

Data supporting the work are contained within the article or Supplementary Materials. Any other raw data are available on request from the authors.

## Supplementary Materials

The following supporting information can be downloaded at: https://www.mdpi.com/article/10.3390/nano14151298/s1, Figure S1: Survey XPS spectra for all samples.
