# Supplementary material for: Enhanced Dye Adsorption on Cold Plasma-Oxidized Multi-Walled Carbon Nanotubes: A Comparative Study
Source: Nanomaterials (Basel). 2024 Aug 1;14(15):1298. doi: 10.3390/nano14151298 (PMC11314139; doi:10.3390/nano14151298)
Supplement: Supplementary file 1 [file nanomaterials-14-01298-s001.zip › nanomaterials-3112263-supplementary.pdf]

Supplementary Data

Enhanced Dye Adsorption on Cold Plasma-Oxidized Multi-Walled Carbon Nanotubes: A Comparative Study

Anastasia Skourti [1,2], Stefania Giannoulia [1], Maria K. Daletou [2,*] and Christos A. Aggelopoulos [1,]**

[1] Laboratory of Cold Plasma and Advanced Techniques for Improving Environmental Systems, Institute of Chemical Engineering Sciences, Foundation for Research and Technology Hellas (FORTH/ICE-HT), 26504, Patras, Greece
[2] Laboratory of Advanced Materials and Electrochemical Energy Conversion Devices, Institute of Chemical Engineering Sciences, Foundation for Research and Technology Hellas (FORTH/ICE-HT), 26504, Patras, Greece

* Correspondence: riadal@iceht.forth.gr; Tel.: +30 2610965213
** Correspondence: caggelop@iceht.forth.gr; Tel.: +30 2610965205

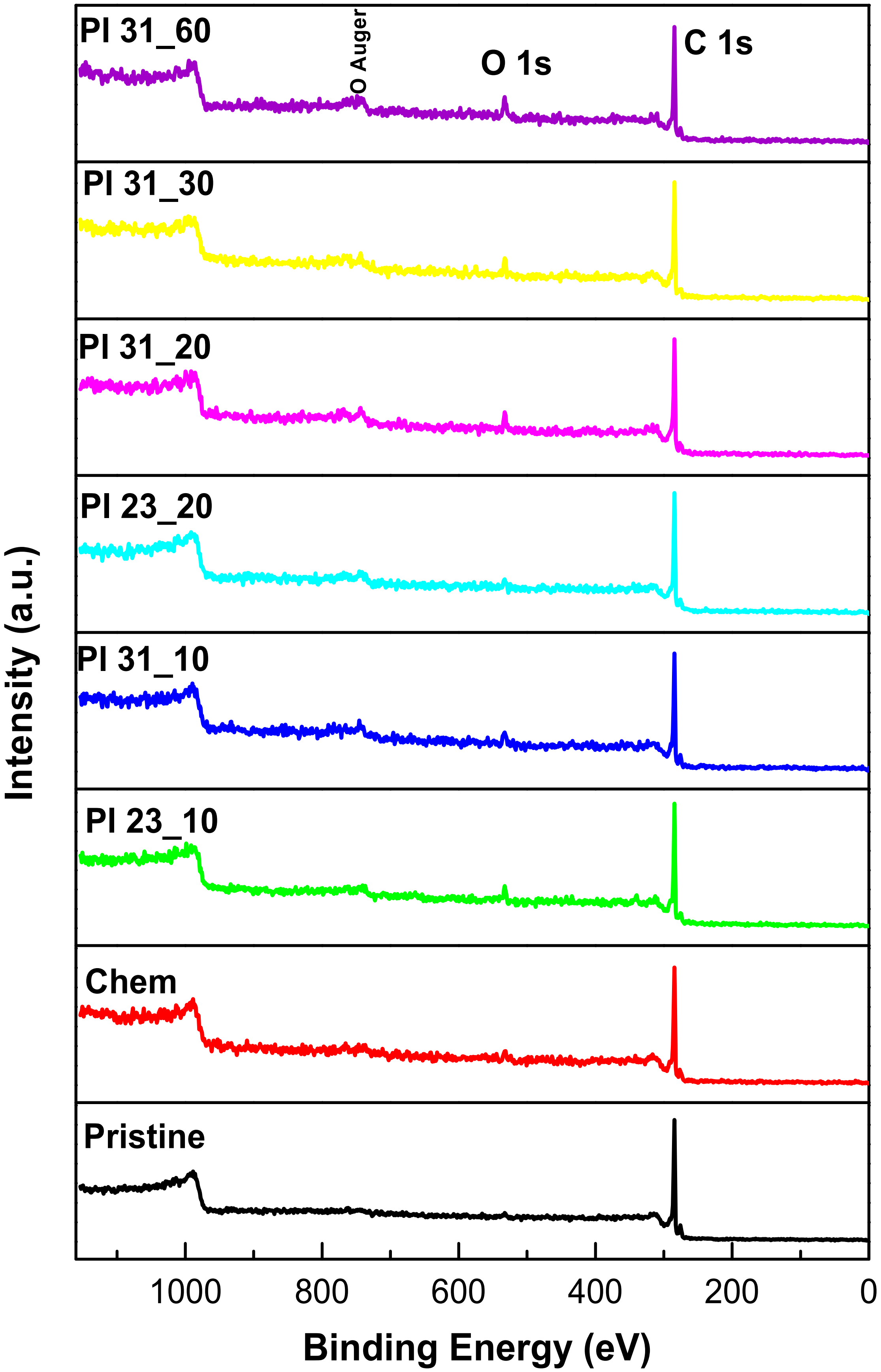

Figure S1. Survey XPS spectra for all samples.
